# Effect of surface roughness and nano-asperities on bacterial–substrate interactions of PHF-PDMS‑modified substrate through nanoarchitectonics

**DOI:** 10.1080/14686996.2026.2751772

**Published:** 2026-10-22

**Authors:** Sorna Lakshmi Anbu, Karthick Velu, Novi Dwi Widya Rini, Harapriya Mohapatra, Subhenjit Hazra, Katsuhiko Ariga, Lok Kumar Shrestha

**Affiliations:** a Centre for Ocean Research, Sathyabama Research Park, Sathyabama Institute of Science and Technology, Chennai, India; b Research Center for Materials Nanoarchitectonics (MANA), National Institute for Materials Science (NIMS), Tsukuba, Ibaraki, Japan; c School of Biological Sciences, National Institute of Science Education and Research, Jatani, India; d Homi Bhabha National Institute, BARC Training School Complex, Mumbai, India; e Centre for Nanoscience and Nanotechnology, Sathyabama Institute of Science and Technology, Chennai, India; f Department of Advanced Materials Science, Graduate School of Frontier Sciences, The University of Tokyo, Kashiwa, Chiba, Japan; g Department of Materials Science, Institute of Pure and Applied Sciences, University of Tsukuba, Tsukuba, Ibaraki, Japan

**Keywords:** Surface mechanics, nanoarchitectonics, AFM, bacterial adhesion, surface roughness

## Abstract

In the present study, we have investigated the role of surface roughness influencing bacterial adhesion as a key to inhibit catheter‑associated infections. Polydimethylsiloxane (PDMS) substrate was fabricated through the incorporation of polyhydroxy fullerenes (PHF) to induce nanoscale roughness. Surface characteristics of developed PHF-PDMS surfaces were carried out using bio-Atomic Force Microscopy (Bio-AFM), along with fluorescent staining–based adhesion analysis for *Staphylococcus* (cocci) and *Klebsiella* (rod-shaped) bacteria. PHF-PDMS exhibited significant nanoscale heterogeneity, reflecting through its altered roughness, and asymmetric height profiles influencing bacterial morphology. Bacteria grown on the PHF-PDMS substrate showed characteristic protruding and membrane morphological changes. Fluorescence imaging corroborated with other findings, showing reduction in bacterial growth, and spatially fragmented adhesion over PHF-PDMS compared to the control surface. Collectively, this study highlights that surface nanoarchitectonics offers antibiotic-free strategies for preventing biofilm formation on biomedical devices.

## IMPACT STATEMENT

Surface modification was employed to prevent bacterial adhesion, as a baseline for antibiofilm coating. Our findings conclude that bacteria-to-substrate nanofeature is the key to formulate antibiotic-free coating applications.

## GRAPHICAL ABSTRACT

**Figure:**
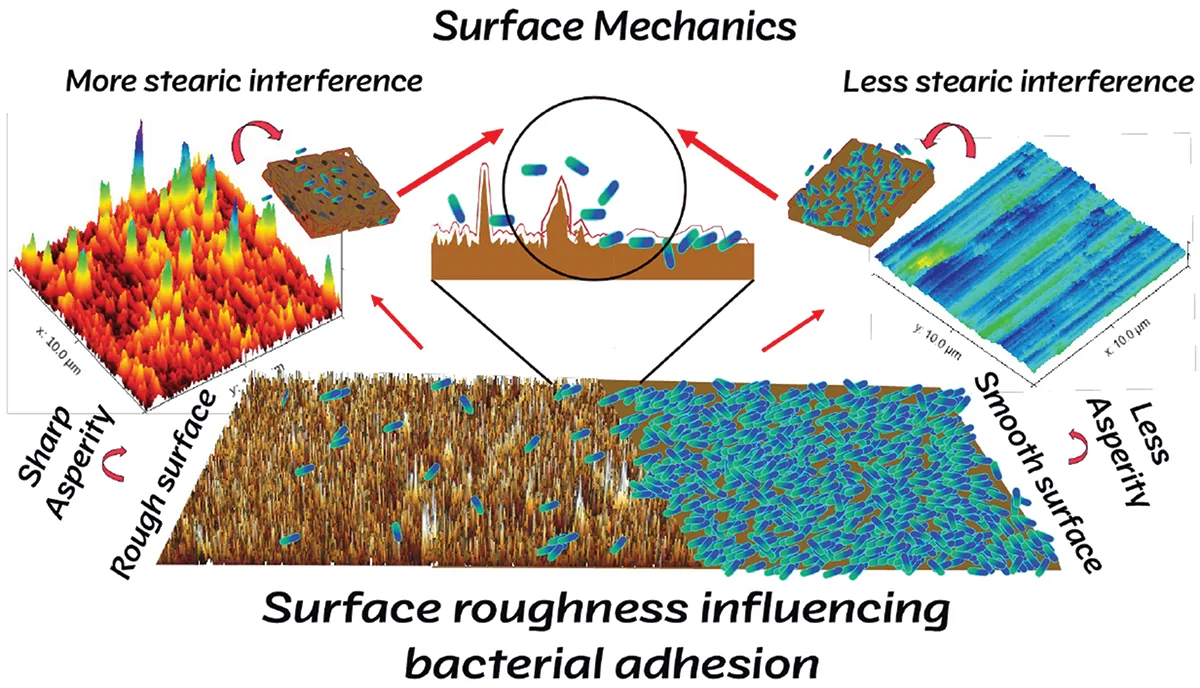

## 1. Introduction

Bacterial adhesion to biomaterial substrates significantly influences the medical sector, especially in critical care units and prosthetic applications. Biofilm formation on implants and catheters affects patient prognosis, leading to secondary infections and increased morbidity. In particular, systemic sepsis for hospitalized patients counted as 25%, with urinary tract infection contributing to incidence of 13,000 cases annually [1]. Due to the prevalence of antimicrobial resistance, treatment for biomaterial–associated infection often results in catheter replacement and surgical intervention, thereby elevating the risk of nosocomial infection [2]. In general, biofilms are complex mixture of exopolysaccharide, genetic matter and proteins embedded within a 3D microbial matrix [3]. Transition of bacteria from planktonic Free State to a sessile state involves series of stages including (I) bacterial interaction with the substrate microenvironment, (II) bacterial flagellar sensing the nanoscale intermolecular forces of substrate, (III) translocation of bacteria from bulk fluid to the substrate, and (IV) Molecular contact and bacterial appendages adhesion [4]. This process is influenced by free energy available in the microenvironment, including van der Waals forces, electrostatic attraction etc. Therefore, modulating the micro-niche energy is a key for developing anti-adhesive substrates to influence molecular-substrate interactions [5]. In this context, numerous investigations on the development of biomimetic and naturally derived polymeric surfaces were implemented against biofilm bacteria showing reduced adhesion [6–8]. Various strategies, including synergistic methods, natural polymers, superhydrophobic coatings, functionalized surfaces, bio-derived nanoparticles, etc., have been demonstrated [9–12]. Among these, implementation of physical barriers for biofilm reduction has shown a sustainable approach and foreseen future [13,14]. Subsequently, bio-inspired polymers are being explored, focusing on nanoscale imprinting or modified patterned surfaces to reduce bacterial adhesion and colonization along with other environmental applications [15].

Tuneable hydrophobic surfaces and patterned substrates are well known to limit contact area and interfacial energetics. Such as, steric interference, reduced Van der Waals interaction affects bacterial attachment, making it kinetically unfavourable. Antibacterial polymers were used to suppress bacterial colonization; for instance, copolymers of glycosylated β-peptides and chitosan-based composites demonstrated reduced bacterial adhesion on matured biofilms [16]. In some cases, several of these polymers exhibit reduced efficiency in physiological conditions due to degradation [17,18]. To address these limitations, polymer systems possessing dual inhibition via hydrophobicity and embedded biocidal agents were used to reduce antibiotic dependence and allow re-sensitization of bacterial species [19]. Furthermore, superhydrophobic surfaces with enhanced roughness can trap interstitial air, generating Laplace pressure that exceeds adhesive forces, thereby weakening cell‑to‑surface interactions and increasing separation between bacterial cells and the substrate [4,20]. Topography modifications rather than the functional modifications also gained significant attention. In recent years, advancements in 3D printing have enabled development of surface architectures incorporating pili-like nanopillars, which promote bacterial clearance [21]. Notably, studies by Ivanova et al. showed bactericidal activity arises from membrane deformation and stretching induced by high magnitude peaks rather than direct penetration of nanopillars, highlighting an elasticity-driven mechano-bactericidal mechanism [22]. In continuation with this, we aim to develop an anti-bacterial surface by incorporating carbon nanoparticle – polyhydroxy fullerenes (PHF) – fabricated into the polydimethylsiloxane (PDMS) polymer as a dual inhibitor of bacterial biofilm formation. PDMS biopolymer was chosen for its biocompatibility and hydrophobicity as a first‑line defence towards bacterial adhesion [23,24]. Similarly, PHF were incorporated into PDMS as an antibacterial agent, these hydrophilic carbon nanoparticles analysed to be effective against biofilm‑forming bacterial organism. This formulation acts as a dual inhibitor to suppress the bacterial proliferation of few adhered cells [25,26]. Incorporated PHF modifies surface parameters of PDMS-elastomer, characterised by SEM, and AFM analysis. Topographical imaging was performed to elucidate surface roughness and higher-order statistics of substrates. Substrate-bacterial interaction was analysed using biofilm-forming bacterial species and evaluated for adhesion under bio-AFM imaging and fluorescent staining.

## 2. Materials and methods

Fullerene (99.5%) and tetrabutylammonium hydroxide were purchased from TCI, India. APTES ((3-Aminopropyl) triethoxysilane) was purchased from Merck, Germany. All other solvents were purchased from SRL, India. SYLGARD™ 182 Silicone Elastomer Kit was purchased from Corning DOW, US and used for the fabrication of PDMS substrate. MTCC strains of *Staphylococcus aureus*, and *Klebsiella pneumoniae* were procured from the CSIR-IMTECH, Chandigarh, India. Nutrient agar, Mannitol Salt Agar (MSA), and other microbial reagents used were purchased from SRL, India.

### 2.1. Synthesis of polyhydroxy fullerene (PHF)

Polyhydroxy fullerene (PHF) was synthesized using hydroxylation protocol adapted and modified from [27,28]. Briefly, 80 mg of fullerene was refluxed with the 60 mL of benzene (all the reactions were performed in a certified fume hood to eliminate inhaling vapours), stirred for 30 mins, yielding a dark purple solution. A 2 mL of 2.5 M sodium hydroxide was added, followed by immediate addition of 0.3 mL of 40% tetrabutylammonium hydroxide (phase transfer catalyst) to benzene when the temperature reached slightly over the boiling point. The mixture underwent phase transition in room temperature, followed by decantation. Dark bottom phase was neutralized using 20 mL of deionized water and stirred for 27–30 h for complete hydroxylation. Product was filtered using Whatman filter paper no. 21 and dried under vacuum at 60°C for 24 h. The obtained crude was washed thrice with methanol, dried under vacuum, stored at 4°C for further experiments. PHF was subjected to XPS analysis by K-Alpha X-ray Photoelectron Spectrometer, Thermoscientific, the Netherlands, with photon energy of monochromatic radiation of Al-kα to determine successful conversion and to analyse its purity, followed by SEM analysis in FEI-Quanta FEG 200F, Thermo Fisher Scientific.

### 2.2. Incorporation of PHF into Polydimethyl siloxane (PDMS)

PHF-incorporated PDMS coatings were fabricated through a drop-casting method, references from [29,30]. In brief, the PHF were pretreated and oven-dried to eliminate residual moisture. PDMS mixture was prepared at an elastomer-to-curing agent in ratio of 10:1 (w/w), mixed under vacuum conditions to minimize air entrapment, and sonicated in ice-cold environment at an intervened pulse mode. Prior to fabrication, PDMS was plasma‑treated for 5 min, followed by immersion in 2% of APTES (dissolved in ethanol) for 30 min to achieve free -NH_2_ bond. To this mixture, PHF was added in a concentration of 1%, followed by additional sonication to achieve uniform dispersion. Mixture was then casted onto the pretreated glass slides of 1 × 1 cm. For the control sample, addition of PHF step was eliminated, and pristine PDMS was coated onto the clean slide followed by drying. Prepared blocks were imaged in SEM by FEI-Quanta FEG 200F, Thermo Fisher Scientific. Further, XRD analysis was carried out to check the incorporation of PHF into PDMS in ARL EQUINOX 3000, Thermo Scientific. In addition, the wetting behaviour of blocks were experimented using sterile water and phosphate-buffered saline (PBS) onto the substrates. The static contact angle (CA) of water and PBS on the substrates was determined using a goniometer (Ram´e-hart, U.S.A.) equipped with DROP image software.

### 2.3. Topographical analysis of polymer roughness under AFM

Bio-AFM was carried out to evaluate mechanical properties and surface topography of PHF-PDMS. AFM measurements were conducted using JPK NanoWizard 4 XP Bioscience, Bruker, Germany, with protocol references from previous literatures [31,32]. An antimony-doped silicon cantilever with a nominal spring constant of 37 N/m and a rectangular probe with 7 nm tip radius, suitable for force measurement of 0.01–0.025 ohm-cm, was carefully mounted onto the cantilever with the help of sterilized tweezer. Prior to analysis, tip was carefully cleaned via bath sonication in isopropanol for 20 min to remove surface contaminants. All measurements were performed in tapping (AC) mode to minimize sample deformation and ensure accurate surface characterization. Imaging was conducted at multiple positions of the sample to generate surface topography, average roughness, and surface roughness profile. Approach amplitude of 0.05 V, with the setpoint of 20 nm and drive frequency of 304.9 kHz were maintained throughout the experiment, with the Z range were fixed at 15 µm. The sample preparation included slides coated with test samples of 1 × 1 cm. Samples were air-dried and mounted on the UV‑sterilized clean slide, secured with adhesive tape. A scan area of 10 µm × 10 µm was acquired at a scan rate of 1 line s^−1^.

### 2.4. Surface mechanics quantification

Surface mechanics plays a critical role in influencing the soft and composite polymers [33]. In case of elastomeric materials such as PDMS, significant surface deflection may occur upon on probe-surface interaction. In this context, high-order statistics, including skewness, kurtosis, local radii, and curvature were evaluated to measure accurate deflection. For this analysis, topography images of control and PHF-PDMS were taken. From the images, 1 × 1 µm of sub-region at centre, four corners, and four boxes inner to corners have been calculated. These nine sub-regions have been selected so that the calculated area covers the average of whole scanned image. Gwyddion software version 2.0 was used for the analysis. Prior to analysis, raw topographical data underwent standard preprocessing steps, including plane levelling, polynomial background subtraction, and line-by-line correction to eliminate scanning artifacts. Within sub-region, parameters of root mean square roughness (RMS a), standard deviation, Kurtosis (R_ku_), Skewness (R_sk_), etc., and statistical quantities were calculated and validated same in the SPM desktop data processor version 8.1. In addition, prominent spikes were measured to determine local radii of curvature. Average of these spikes reflects on the cumulative local roughness of those particular surfaces. Furthermore, relationship between the measurement of lateral extension vs surface elevation, reflecting on the asperity of the spikes was examined to assess surface protrusions.

### 2.5. Encapsulation of PHF

Encapsulation and *in vitro* release studies were carried out to evaluate the availability and release behaviour of PHF from PHF–PDMS formulations, following the previously reported methodologies [34]. In brief, concentration equivalent to PHF-PDMS were encapsulated using a co-axial pumping system. A controlled flow rate was maintained using a syringe setup to obtain pellets of uniform size. The distribution of PHF on the pellets was confirmed using UV–visible spectroscopic analysis. Prepared pellets were subsequently dried in a vacuum oven at 45°C for 20 min. For *in vitro* release studies, dried pellets were sealed in a dialysis membrane (molecular weight cut-off: 20 k Da) and immersed in PBS under control conditions. At time intervals of 1–6 h, followed by 12, 24, and 48 h, aliquots were analysed using UV–visible spectroscopy to quantify PHF release. The cumulative release profile of PHF into PBS was calculated and plotted as a function of time to determine release kinetics.

### 2.6. PHF release kinetics

*In vitro* release data of PHF from spherical pellets were analysed to elucidate release mechanism. To determine the therapeutic activity of PHF, a diffusion‑based kinetic approach was adapted from a previous article [35]. At predetermined time intervals of every 1 h till 48 h, UV-Visible absorbance maxima were recorded to quantify the amount of PHF released into the medium. The concentration of PHF at each time point was determined using a formula of the Beer–Lambert law:

A=ε.C.L

A = absorbance, ε = molar absorptivity, C = concentration, L = path length of the cuvette.

Calculated concentrations at different time intervals were used to determine relative cumulative percentage at different time intervals by following formula:

$$\%\mathrm{of}\;\mathrm{release}=\left(\mathrm{Ct}/C\;\mathrm{total}\right)*100$$

Ct – Concentration at different time, C total – total concentration.

To plot kinetic modelling, root of time, log of time (log_t_), and log of percentage (log_%_) release were calculated. Release data were then fitted with curve models and linear regression models using OriginPro learning edition. To know the best‑fitting model, the cumulative percentage release was plotted against time and analysed with models of linear fitting, including zero-order kinetics, first-order kinetics, Higuchi model, and exponential. Non-linear fitting models have also been experimented to check the best fit. Regression coefficient (R^2^) and adjusted *R* values were compared along with the residual sum of squares. Furthermore, Pearson R was calculated, to determine the dominant transport model for this PHF-PDMS formulation. The best‑fitting curve was selected and taken as standard for further antibacterial studies.

### 2.7. Maintenance of microbial culture

*Staphylococcus aureus, and Klebsiella pneumoniae* cultures were purchased from the Microbial Type Culture Collection (MTCC), Chandigarh, India. These micro-organisms were selected based on the criteria of:

1. Clinical relevance: active biofilm organism and
2. the morphology i.e. Gram-positive cocci (round) and Gram-negative bacilli (rod).

Different morphology advertently helps in analysing the surface mechanics that pertains to shape-dependent adhesion onto incorporated surface. Lyophilized culture was aseptically inoculated in the distilled water, as advised by the MTCC manual, and maintained in streak plates of respective selective media. Prior to experiment, cultures were adjusted using McFarland standards to maintain a final concentration of 5 × 10^8^ CFU/mL [36]. From prior experiment (CV assay) different concentration of PHF, ideal concentration was selected for further studies (Figure S1 and Figure S2: Supplementary Information). For control samples, the bacterial suspension of 10 µL was drop-casted onto sonicated cleaned PDMS coverslip, followed by graded ethanol dehydration with intermittent PBS wash. Dehydrated coverslips were dried in the cabinet desiccator to remove residual moisture. For test conditions, PHF-PDMS slabs were prepared at 1 × 1 cm and immersed in the broth overnight to facilitate adhesion, followed by PBS wash and drying. The prepared coverslips and slabs were mounted onto the clean glass slides and stored at room temperature until AFM screening.

### 2.8. Biofilm analysis on treated bacterial species under AFM imaging

Morphological alterations induced by PHF-PDMS on biofilm were studied using Bio-AFM analysis on JPK NanoWizard 4 XP Bioscience, Bruker, Germany [37]. Imaging was performed in AC mode, using a cantilever of spring constant 3 k/s, on a silicon‑coated wafer of 10 × 10 µm size with a resolution of 512 × 512 pixels. Average roughness along with the length and histograms were recorded. The Z range was maintained at 15 µm with a tip velocity of 2.27 µm/s and line rate of 0.35 Hz. Quantitative surface parameters, including average roughness, and height distribution histograms, were extracted from the topographical data. In addition to cell geometry influence over adhesion, individual bacterial cells were focused and topographed to analyse PHF influence over cell membrane damage.

### 2.9. Visualization of substrate adhesion against bacterial biofilm

Biofilm quantification was assessed by acridine orange fluorescence staining following literature [38]. Negative replica staining was performed for test and control samples. Briefly, overnight cultures were incubated with PDMS and PHF-PDMS samples for overnight. A 0.01% acridine orange solution was utilized to stain the biofilm. Control and all other triplicates were negatively replicated onto a clean glass slide and imprinted with weight for 4 h in the incubator. All slides were washed twice with PBS to remove the suspending cells, and acridine orange was flooded onto the target site, followed by dark incubation for 30 min. After incubation, excess stain was removed by washing with PBS, and the samples were vacuum‑dried. Fluorescence microscopy was carried out in LSM 900, Carl Zeiss, Germany. The stained samples were mounted on clean glass slides and visualized under oil immersion to assess biofilm distribution, surface coverage, and adhesion patterns.

### 2.10. Statistical analysis

Experiments were carried out in triplicates, and data were expressed as mean ± standard error. OriginPro Learning Edition from OriginLab, US, was utilized for the graph preparation and curve fitting analysis.

## 3. Results and discussions

Interaction between bacteria and substrate is primarily governed by the physiochemical parameters of surface, including roughness, skewness, surface energy, and asperity, etc [35]. Herein, surface roughness was applied as a nano‑architectonic approach to induce bactericidal activity. PDMS were employed as a base polymer, and PHF was incorporated, markedly influencing the bacterial interaction and subsequent biofilm formation. Surface characteristics indicated an increase in roughness, asperity, and curvature. Furthermore, increased local roughness attributes to morphological deformation of adhered bacteria, and detailed discussion on the surface influence over bacterial-substrate interaction presented in the following section:

### 3.1. Synthesis of PHF

For the synthesis of PHF, tetrabutylammonium hydroxide acted as a phase transfer-catalyst, facilitating nucleophilic addition of a hydroxyl group to fullerene cage (Figure 1(a)). Following hydroxylation, modified fullerene (PHF) reduced into sediments and remained undissolved in benzene. Resulting PHF was obtained as a light yellow to brownish solid and demonstrated good solubility in distilled water without observable aggregation. The synthesized PHF exhibited distinct physical characteristics compared to pristine fullerene in terms of both colour and texture. Change in colour exhibited by PHF is different from fullerene, which is a characteristic confirmation of fullerene conversion as reported in previous studies [27,39]. Additionally, PHF synthesized via alkali-assisted and phase-transfer catalytic routes has been reported to exhibit a brown coloration and complete solubility in water [27,40].

**Figure 1.**
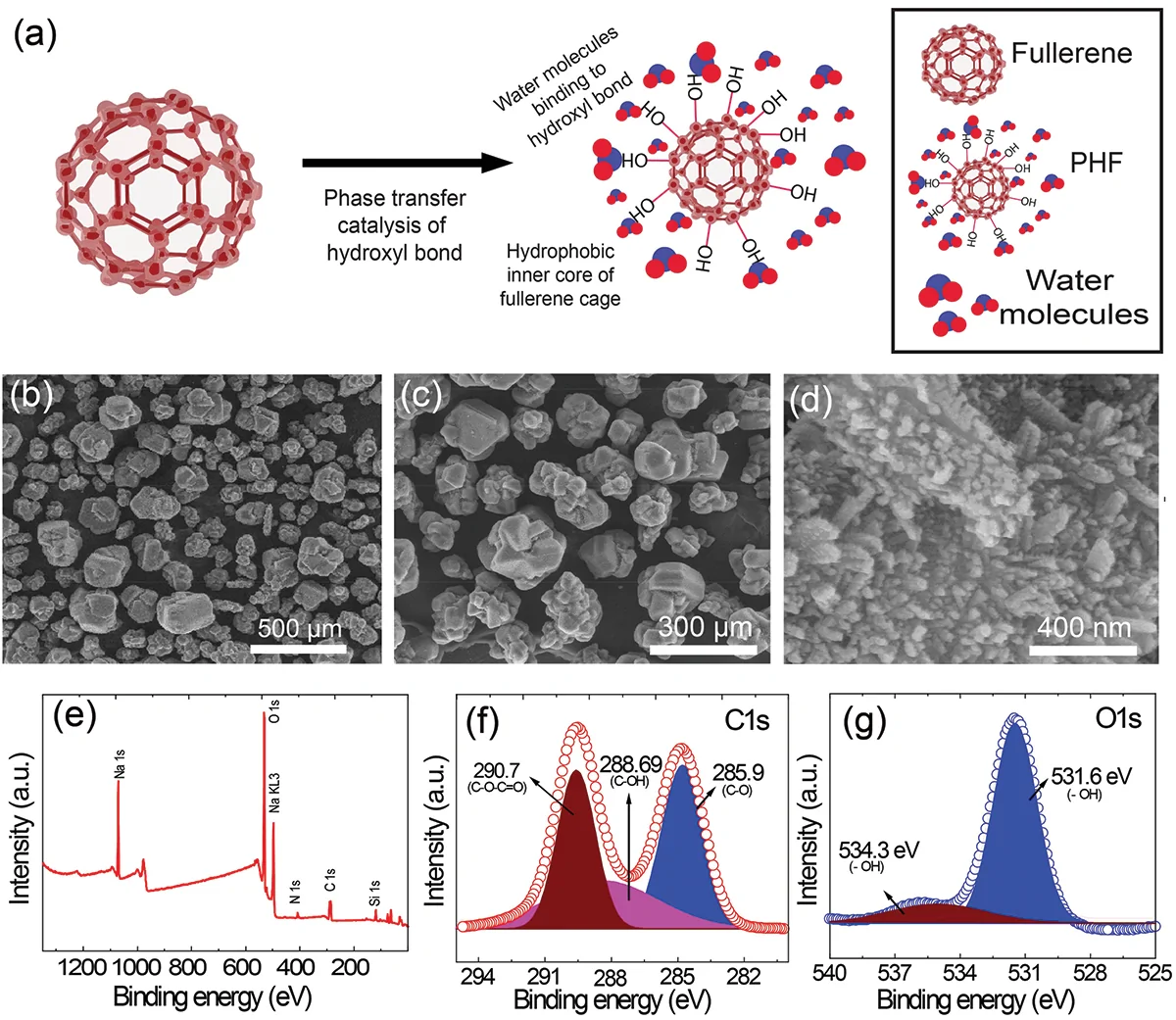
Schematic representation of the structure of PHF and synthesis of PHF: (a) SEM micrographs of PHF; (b) - (d) aggregates; (e) XPS survey spectra; (f) corresponding C 1s and (g) O 1s spectra with deconvoluted peaks of PHF, respectively.

The SEM micrography of PHF revealed small aggregates of PHF of varying size (Figure 1(b–d)), suggesting aggregation behaviour and intermolecular clustering. Survey spectra of XPS confirmed the presence of Carbon, Oxygen, Sodium, and no other significant impurities (Figure 1(e)). Elemental analysis of O1s spectrum confirmed the presence of a hydroxyl (−OH) functional group in PHF, attributing to cage hydroxylation (Figure 1(g)). In the C1s spectrum, the presence of two peaks of PHF is due to the C1s shift by broadening of C-OH bond, confirming the incorporation of more hydroxyl functional groups. This is in consistent with previously reported XPS spectra of the fullerenol and oxygenated fullerene [40,41].

XPS analysis of PHF exhibited characteristic C1s peaks at 290 eV and 285 eV (Figure 1(f)) corresponding to the C-OH and O-C=O bonds, which differ from the pristine fullerene peak observed at 284 eV, respectively. In the O1s spectrum, at Sp^2^ level peak maxima was attributed to 531 eV, corresponding to the hydroxyl bond (C-OH). This peak position differs from the higher binding energy peak around ~533 eV, typically associated with physically adsorbed oxygen rather than chemically bonded hydroxyl groups. The observed shift toward lower binding energy, therefore, supports the formation of chemically bonded C–OH groups in PHF [27,39]. Furthermore, oxygen atomic percentage was found to be around 44%, indicating high degree of hydroxylation onto fullerene cage.

### 3.2. Fabrication of PHF assemblies incorporated PDMS

PDMS slabs were prepared and used as controls, while PHF incorporated in PDMS were fabricated and imaged in SEM micrographs (Figure 2(a–c)). The microfeatures of the blocks were examined along with the thickness. PHF on PDMS were initially bonded by -OH of PHF to CH_3_ of PDMS. Furthermore, PHF-PDMS drop-casted slabs exhibited significant dispersion around 100°C, as PDMS behaves as an elastomeric matrix, causing good dispersion of surface PHF embedded onto the PDMS [43,44]. The PHF synthesized tends to aggregate, displaying a coarse appearance. Therefore, a curing condition of 100°C under 8 Pa vacuum for 20 h was determined to be optimal, providing the required stretchability of PDMS while ensuring adequate dispersion of PHF within the PDMS matrix.

**Figure 2.**
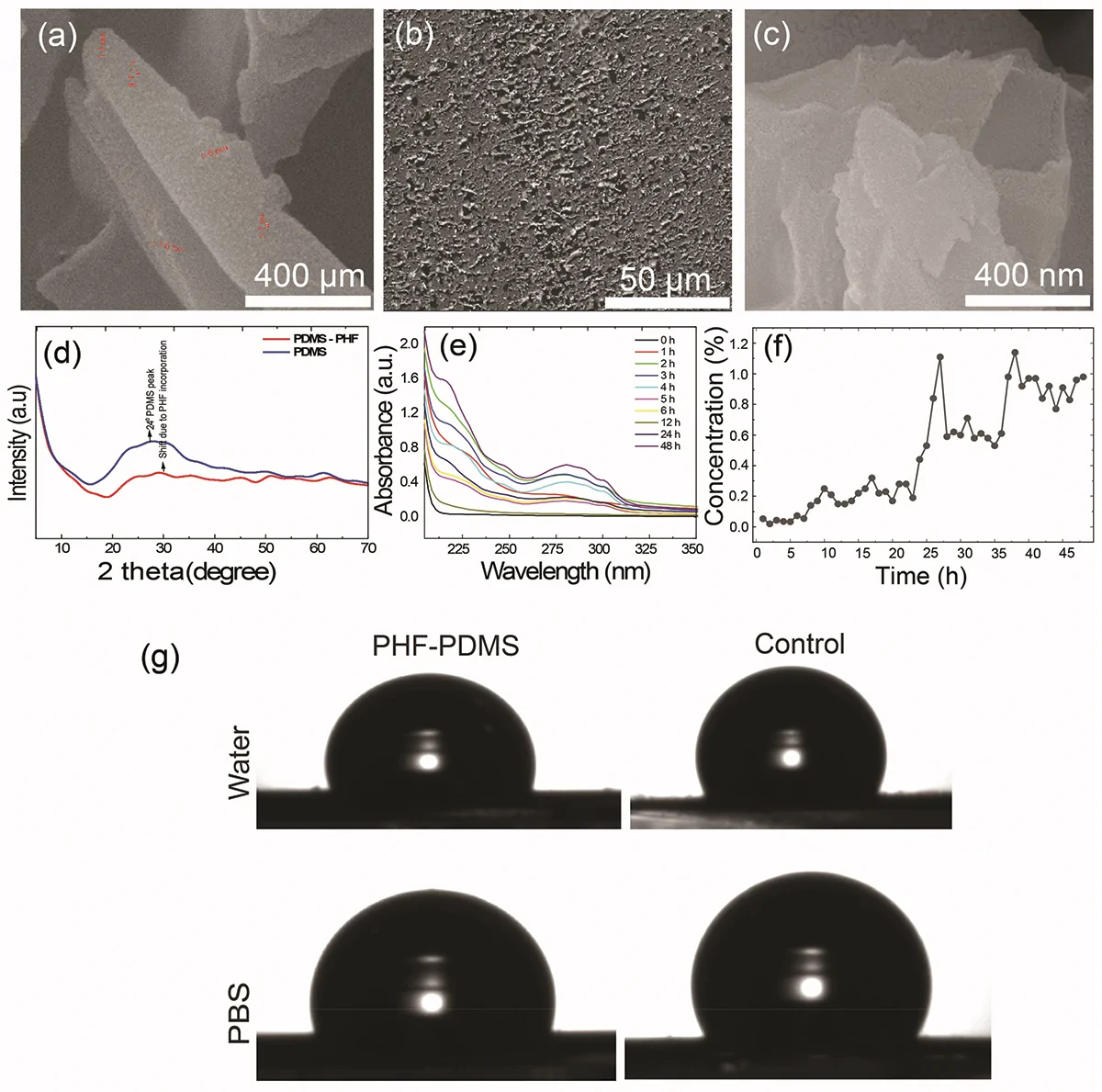
SEM imaging of PHF embedded in a PDMS matrix (a) - (c), (d) XRD spectra of PHF-PDMS and pristine PDMS, (e) Release vs. concentration of PHF from PDMS, (f) % concentration release of PHF at regular intervals for 48 hours, and (g) Contact angle measurement taken on sterile water and PBS substrate through the sessile drop technique.

Further to dispersion and incorporation of PHF, the PHF-PDMS were examined using SEM analysis to analyse microfeatures and aggregation behaviour of PHF in PDMS (Figure 2). In Figure 2(a) shows PDMS coated with PHF, showing bigger aggregates covering the entire surface area with a spiky appearance. Figure 2(b,c) shows PHF‑incorporated PDMS having a less slithering appearance, with smaller aggregates evenly dispersed across the surface, with aggregate sizes ranging from approximately 6 to 15 nm, which are in accordance with earlier report [42,45]. Since PDMS is an amorphous polymer, no sharp diffraction peaks were observed in the XRD pattern (red line, Figure 2(d)). A broad characteristic peak of PDMS was observed at around 24°, consistent with previously reported studies [46,47]. Upon incorporation of crystalline PHF (blue line), additional low-intensity peaks appeared in the lower 2θ region, indicating the presence of crystalline PHF domains within the PDMS matrix. A slight shift in the characteristic peak of PDMS was also observed, confirming the incorporation of PHF into PDMS matrix [48]. Surface wettability studies were carried out to analyse the hydrophobic behaviour of PDMS after PHF incorporation (Figure 2(g)). Upon analysing, PDMS showed more hydrophobicity towards PBS followed by water with the contact angle of 116.30 ± 0.01° and 111.52 ± 0.01°, respectively (Table 1). Similarly, after incorporation of PHF, the range of contact angle has shown slight decrease in both PBS and water as 111.52 ± 0.01° and 108.95 ± 0.01°. Despite a slight decrease after incorporation of PHF, the wettability behaviour was found to be remained as hydrophobic [23,49].

**Table 1.** Contact angle of PDMS and PHF-PDMS obtained from different solvents such as sterile water and PBS.

S. No	Sample	Contact angle	Surface energy
1	PDMS- Water	111.52 ± 0.01	16.21 ± 0.01
2	PDMS-PBS	116.30 ± 0.01	13.55 ± 0.01
3	PHF-PDMS-Water	108.95 ± 0.01	17.7 ± 0.01
4	PHF-PDMS-PBS	111.52 ± 0.01	16.21 ± 0.01

### 3.3. Release kinetics

Release kinetics were analysed to see the PHF release from PHF-PDMS to check the bioavailability of nanoparticle for exerting its antibacterial activity. Release kinetics are useful to study the toxicity behaviour of nanoparticles, biotransformation, etc [50]. Release behaviour was examined using UV- Visible spectroscopy, demonstrating a sustained release profile for the period of 48 h (Table S1: Supplementary Information). During the initial stage of first 6 h, PHF exhibited a slow gradual release (Figure 2(e)), followed by multiphase fluctuating release behaviour. This may attribute towards PHF embedding onto PDMS matrix rather than superficially adsorbed. An increased peak in release was observed around 20–27 h, which may indicate increased hydration of matrix with PHF release. This was followed by a small transient phase of reduced release around period of 28–33 h (Figure 2(f)). This fluctuating release pattern indicates involvement of heterogenous transport process rather than simple diffusion. Further, to quantify released concentration, curve fittings were plotted using the Beer-Lamberts Law.

Initial concentration was found to be 8.87 × 10^4^ Mol/L at 0 h before diffusion starts. Measured concentration at 1st h was very minimal, indicating that diffusion just initiated. Nature of the release were continuous increasing till 6 h, followed by fluctuation in release concentration till 25 h. Again at 25 h, peak in release was observed, which is also reflected in the % of concentration released.

These observations also support that the dominant transport mechanism involves heterogenous transport rather than simple diffusion. To further characterize release kinetics, time-versus-concentration data were fitted using OriginPro learning edition. Initially, the time vs concentration graph was plotted showing the non-linear curve fitting. Both linear and non-linear curve fitting models were evaluated and compared using correlation coefficient (R), adjusted R^2^, and residual sum of squares (RSS) for first‑order, Higuchi, zero‑order, Korsmeyer- Peppas. Among the tested models, the Korsmeyer-Peppas provided the moderate to good fit with an *R* value of 0.8515, indicating a transport-II release behaviour, fitted using the formula of:

$$\log\left(\frac{M_{t}}{M\infty }\right)=\log k+n\log\left(t\right)$$

Followed by moderate fit with the Higuchi model of R^2^ with 0.78, suggesting that PHF release occurs through a complex mechanism involving diffusion coupled with matrix-related transport phenomena. The sustained release observed up to 48 h indicates the potential of the PHF–PDMS system for prolonged antimicrobial activity. Under a biological environment, PDMS‑doped matrix diffusion depends on the balance between hydrophobic interactions and hydrate effects, leading to non-exponential diffusion profile. In the present study, fluctuation behaviour attributes to the diffusion and partitioning of PHF from the polymeric matrix, influenced by its heterogeneous distribution and interaction to the aqueous release medium, same were confirmed in topographical analysis. The present results are consistent with previously reported studies on fullerenol and PDMS based delivery systems, which also demonstrated intermittent release behaviour and gradual degradation from the carrier matrix [51–53]. Releasing of PHF from PDMS confirms reversible bonding of it to substrate, thereby confirming the bioavailability of PHF in environment. There are several studies which focuses on the hydrophilic properties of PHF and its crucial role in acting as ROS scavenger in mammalian environment aiding in neuro protection and radio protection of epidermal activity [54,55]. However, further studies are needed to stabilize the concentration of PHF in environment.

### 3.4. Topographical analysis of PDMS slab under AFM

Bio-AFM imaging was conducted to characterize the surface topography of PHF incorporated into the PDMS formulation. Analysis was aimed at quantifying alterations in surface roughness parameters and correlating these modifications with bacterial biofilm adhesion behaviour on the substrate. Results indicated that an increase in surface roughness causes a reduction in effective adhesion force between the substrate and the biofilm, thereby limiting bacterial attachment and subsequent biofilm formation [6,56]. Furthermore, both surface roughness and elastic properties of treated and control surfaces were systematically studied (Figure 3).

**Figure 3.**
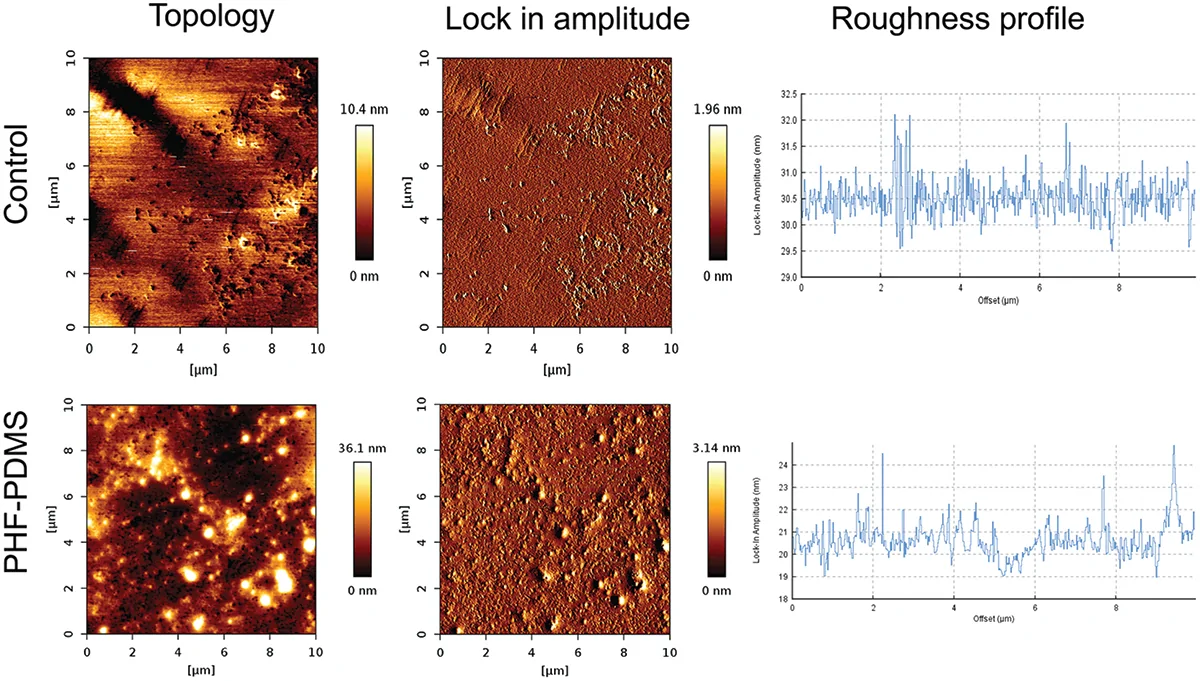
Topographical analysis of PHF-PDMS and pristine PDMS showing morphological modification along with histogram of average roughness.

Topographical analysis of both control and test was captured at a resolution of 512 × 512 pixels. The height measured and lock-in amplitude parameters indicated a significant increase in roughness for PHF-incorporated sample compared to the pristine PDMS. As blocks were coated manually, blebs and sharp peaks were observed in the PDMS, similar to the PHF-PDMS. However, PHF-PDMS slabs exhibited a greater number of broader peaks with more spherical like blebs, attributed towards aggregation of PHF onto PDMS matrix (Figure 3 PHF-PDMS). The peaks varied in sizes (Table 2) and broadened peaks are due to PHF aggregation, which is consistent with the SEM analysis. The height of the peaks was in the range of 15–30 nm in size, indicating reduced contact area than the smoother PDMS slabs.

**Table 2.** Variation in mechanical parameters of PHF-PDMS and pristine PDMS.

Parameters	PDMS (Control)	PHF-PDMS (Test)
Roughness average (Ra)	270.2 nm	524.5 nm
Root Mean Square Roughness (Rq)	387.5 pm	742.2 pm
Peak to Valley Roughness (Rt)	4.88 nm	7.47 nm
Z maxima	33 nm	25 nm
Length of profile	10 µm	10 µm
Average height measured (Y axis)	237.87 pm	143 pm
Average height measured (X axis)	310.33 pm	475 pm

Root Mean Square (RMS) roughness calculated from the root mean roughness also confirms an increase in peak roughness and peak-valley ratio, thereby indicating surface modification of polymer at Z axis. Furthermore, its influence on other higher order statistics will be explained in following.

### 3.5. Influence of surface mechanics of PHF over substrate

Topographical analysis revealed that the roughness of PHF-PDMS has been increased twofold compared to pristine PDMS. In addition to roughness, surface mechanical properties play a critical role in dictating how biological interfaces interact with their material surface. Herein, parameters such as asperity, skewness, and kurtosis were analysed to understand the mechanical interaction between bacterial cells and substrate. Based on this mechanistic approach, we calculated the roughness metrics – Arithmetic Mean Height (Sa) and Root mean square height (Sq), followed by higher-order statistics of skewness and kurtosis. Furthermore, substrate-biofilm interaction was predicted using local curvature and asperity, providing insights on bacterial attachment behaviour. The mean surface roughness was calculated to be 13.99 nm, indicating moderate texturing of PHF-PDMS compared to control (pristine PDMS), exhibited roughness of 2.9 nm. The low roughness value of control sample corresponds to a near-Gaussian surface profile with minimal sharp asperities at the microscale, suggesting a relatively smooth topography with a larger effective contact area available for bacterial adhesion. Comparing the RMS roughness, PHF-PDMS (Sq) distribution revealed that the lower and central-right domains exhibited pronounced surface elevations, representing mechanically active regions. Regions such as the lower-left domain showed comparatively reduced roughness, although still higher than the control sample.

This interprets heterogenous contact mapping across PHF-PDMS, resulting in variation of stiffness and adhesion force. Skewness was observed to be negatively correlated, and asymmetric valleys and depth were observed in control [57]. Comparatively, the PHF-PDMS showed a fourfold increase in skewness, confirming a peak-dominated area at nanoscale, associated with reduced effective contact area for bacterial adhesion and an energetically unfavourable surface for stable bacterial attachment. Further to skewness, asperity was calculated to analyse the sharpness and flatness of peak and nearby region at nanoscale. In this context, kurtosis was calculated for all nine regions of both the control and test sample. For the PHF-PDMS surface exhibited both negative and positive kurtosis values distributed across the surface, confirming heterogeneous spike distribution within the slab, unlike the control sample, which was dominated by broader and flatter surface regions. Collectively, these results indicate reduction in broad area, dominated by sharp peaks theoretically supporting reduced adhesion through mechanical interlocking.

For furthermore analysis on surface mechanics, local curvature and local slope were calculated to quantify the degree of surface bending and gradient at individual asperities. Regions with positive local curvature, corresponding to sharp surface protrusions, generate localized indentation points upon contact with bacterial membranes. This results in a simultaneous reduction in effective contact area and an increase in localized mechanical stress, causing deformation of the bacterial membrane [58]. In consonance with this, a graph was plotted to visualize surface roughness distribution over surfaces of both control and PHF-PDMS (Figure 4). Significant differences were observed for root mean square with median of 9.4 nm and 2.5 nm for PHF-PDMS and control respectively, similarly for the arithmetic mean roughness was calculated to be 7.3 nm and 2 nm. Higher-order statistics, including skewness supported observations on topographical differences. PHF-PDMS showed positive correlation, indicating its protrusion dominated-surface whereas control surfaces were dominated by relatively symmetric, flat valley-like characteristics. Kurtosis distribution plots revealed an extended violin tail for PHF–PDMS, indicating the presence of sharp localized protrusions, while the control surface exhibited a suppressed violin distribution, characteristic of smoother curvature dominated by broader peaks with minimal sharp asperities. These results are in consistent with the previous reports [59,60].

**Figure 4.**
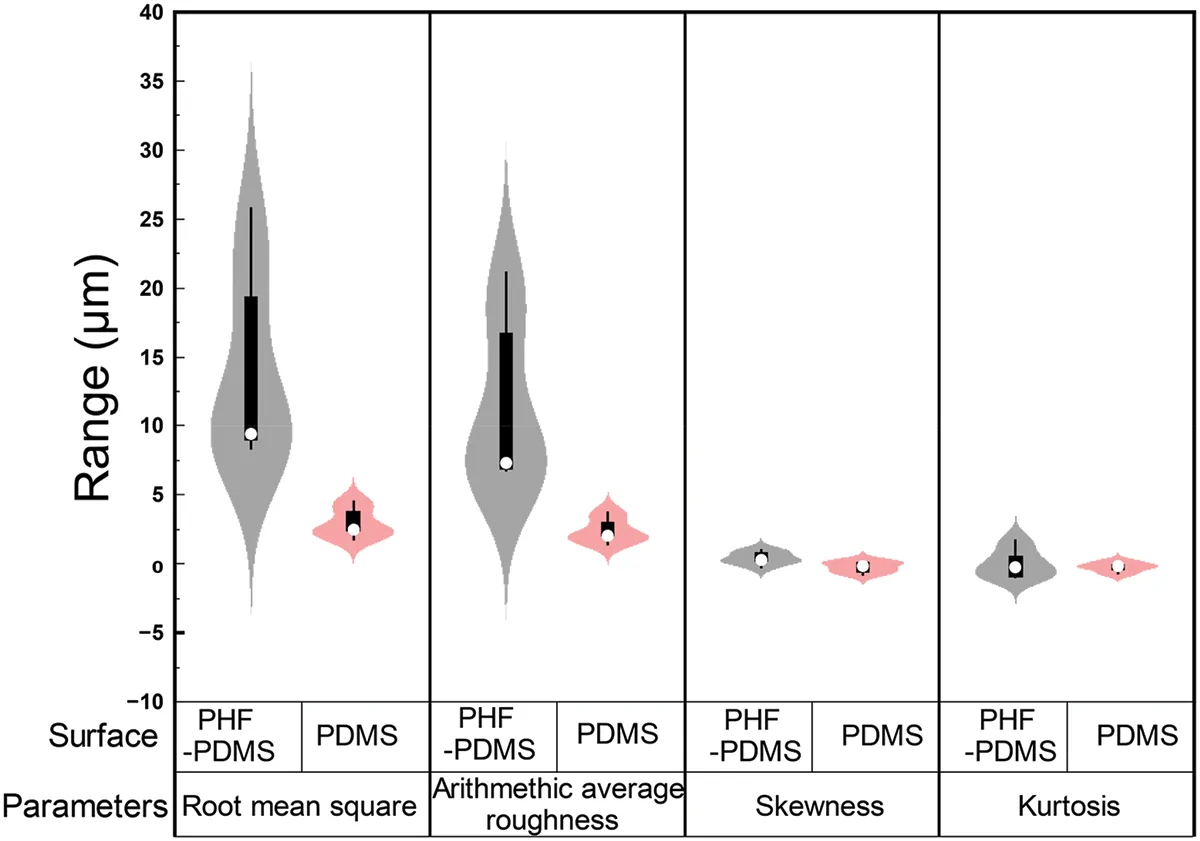
Graphical representation of variation in higher-order statistics between PHF-PDMS and pristine PDMS.

As shown in Figure 5 (Spectral analysis), the asperity index confirmed a higher density of surface peaks on the PHF–PDMS sample compared to the control surface. Spectral analysis on peaks subsequently revealed that asperity–valley distribution was significantly more pronounced in the PHF–PDMS surface, with peak heights reaching up to 350 nm. While the control sample predominantly exhibited valley-dominated regions, typically represented by lower-height features with occasional depressions, and peak heights not exceeding 24 nm. Local radii of curvature, which is inversely related to curvature, was also analysed to understand peak geometry. Smaller local radii correspond to sharper peaks, whereas larger radii indicate broader and smoother surfaces. Surfaces with sharp peaks and smaller radii reduce the effective contact area while increasing localized mechanical stress at the interface, thereby influencing bacterial attachment behaviour. To ensure surface heterogeneity, test samples were topographically analysed at various regions across the surface. Spectral scanning confirmed the presence of sharp peaks and well-defined asperity edges distributed throughout the test surface (Figure 6), indicating that the nanostructured features were not localized but uniformly distributed across the PHF-PDMS landscape.

**Figure 5.**
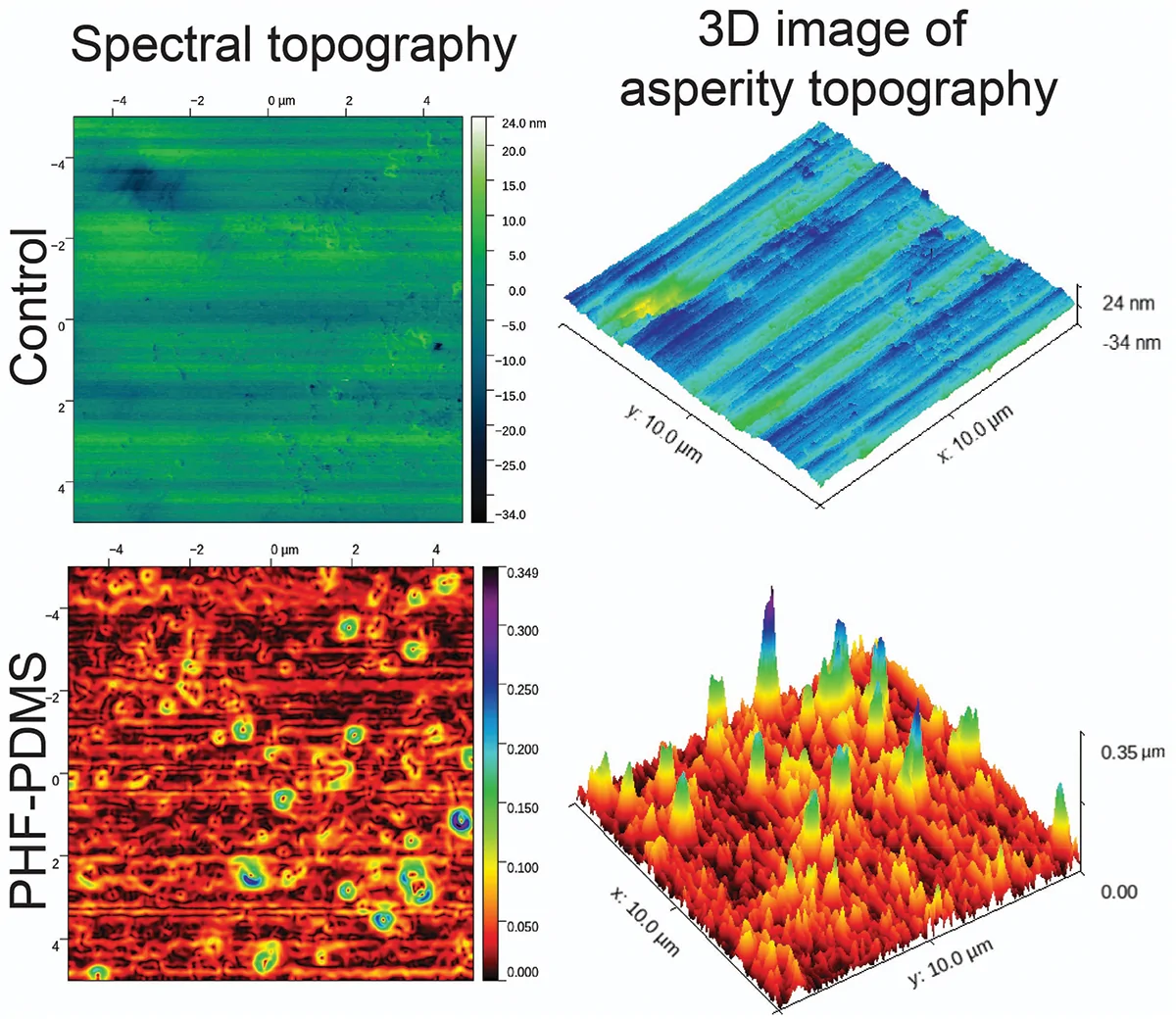
Spectral analysis of peak asperities exhibited by PHF-PDMS and pristine PDMS in 2D and 3D, showing differences in surface mechanics.

**Figure 6.**
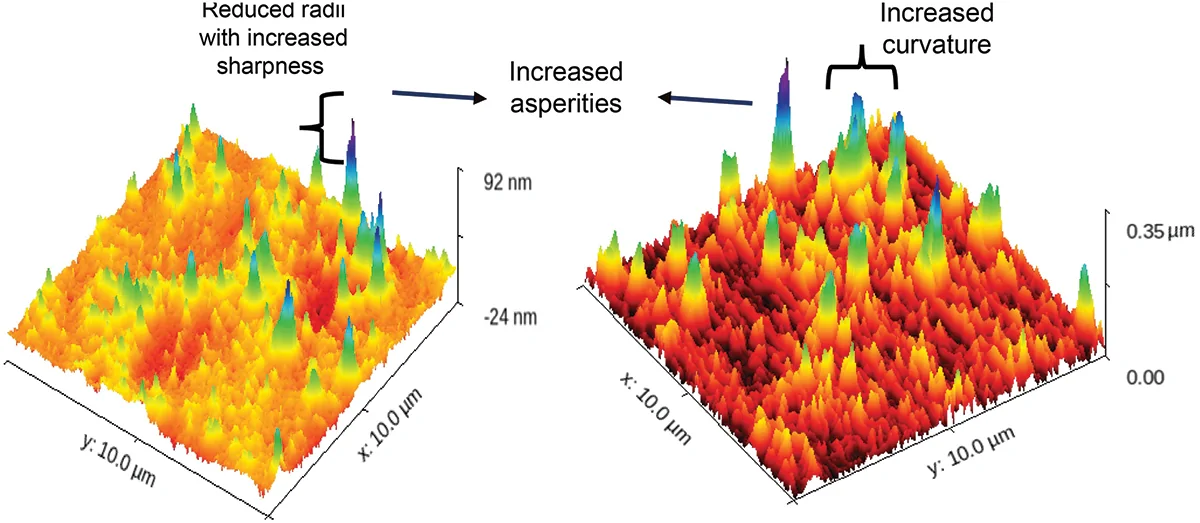
Spectral analysis on PHF-PDMS substrate on various planes depicted in 3D emphasizing on peak properties such as sharpness, radii of curvature and asperities.

The height maxima across different landscapes were measured, and peaks were colour-coded based on the asperity (sharpness) distribution. Analysis of Figure 6 revealed that the asperity distribution is highly heterogeneous, ranging from 92 nm to 3.2 µm, indicating that the incorporated PHF influences the surface topography across both nano- and submicron scales. Such multiscale roughness is known to influence adhesion force and interfacial energy depending on whether the biological entity interacts with nano- or submicron-scale asperities [54]. From asperity distribution of Figure 6, it is evident that the majority of regions in PHF-PDMS are in the range of 40–50 nm (brown regions), followed by varying regions ranging of 80–100 nm (yellow), 140 nm (blue), and 250 nm (dark blue), respectively. Spectral peak analysis was performed to characterize the peak geometry and tip characteristics, which are critical in determining mechanical interaction at the contact interface. It has previously been hypothesized that the alignment and geometry of nanopillars influence interfacial kinetics and can induce protrusion into bacterial cells.

Computational analysis by Velic et al. demonstrated that non-developable, enveloped surface deformations generate significant localized in-plane strain, which can lead to rupture of the bacterial cell wall [62]. Subsequently, experiments on eukaryotic cells revealed that nanopatterned surfaces have a positive correlation on cell proliferation and migration [63]. This observation is particularly relevant as implant, and nanopatterned structures provide reduced toxicity over conventional antibiotics. The surface mechanical parameters for PHF-PDMS revealed increased curvature, roughness, and peak asperity collectively known to reduce contact area and alter energy for bacteria to attach.

### 3.6. Bio-adhesion assay under fluorescent staining

In order to visualize active bacterial biofilm, acridine orange staining was performed to quantify viable bacterial cells. To minimize PDMS autofluorescence, all the images were processed with background subtraction and negative replica visualization. After preprocessing, overall images revealed marked reduction in both adhesion density and spatial continuity on PHF-PDMS compared to control PDMS for both *Klebsiella* (rod-shaped) and *Staphylococcus* (cocci) species. However, the extent and pattern of inhibition varied significantly between two bacterial species. As anticipated, cocci bacteria exhibited extensive biofilm formation after overnight incubation in the control substrate, marked by increased intensity [64].

The 2D fluorescence topography of control sample revealed moderate to high fluorescence intensity, directly proportional to the live bacterial cells, indicating localized colony formation (Figure 7 control). The spatial intensity plot of 3D reconstructed image demonstrated continuous formation of surface colonization in control sample of cocci, whereas for the rod-shaped bacteria exhibited a spike-like spatial distribution, confirming the presence of localized microcolony formation rather than uniform surface coverage. In contrast, the adhesion in PHF-PDMS substrate showed a significantly less dense distribution of fluorescence signal, indicating a reduction in early-stage biofilm formation. The PHF-PDMS substrate is known to exhibit significant inhibitory effect on rod structure than the cocci species. This behaviour is consistent with previous reports suggesting that rod-shaped bacteria are more susceptible to increased membrane stretching and deformation than cocci structures while interacting with nanoscale asperities [65].

**Figure 7.**
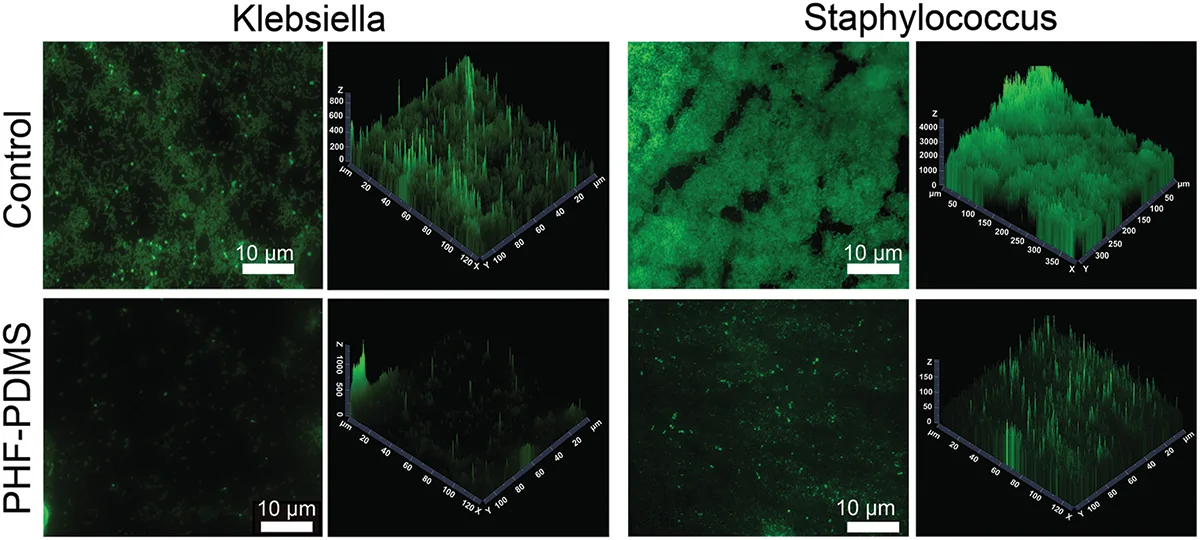
Fluorescent staining, revealing overnight growth and adherence of both bacterial species onto PHF-PDMS and pristine PDMS.

### 3.7. Substrate roughness influence on bacterial morphology

Fluorescent staining clearly indicated that bacterial adhesion was significantly reduced in PHF-PDMS compared to control. To further investigate the influence of surface roughness on bacterial morphology, two bacterial species were incubated overnight on AFM-characterized substrates of PDMS and PHF–PDMS. Following incubation, samples were analysed under tapping mode AFM to evaluate bacterial surface morphology. Overnight incubation was followed to ensure a sufficient time period for both bacterial species to adhere and form biofilm as well as to observe any surface-induced morphological modifications as per previous literature [66]. The topography analysis of *Staphylococcus* on control PDMS revealed a smooth, laterally continuous surface, characterized by low-amplitude height variation. Cells maintained characteristic spherical morphology with smooth exterior, evident from the reduced blue-green zone (Figure 8 (Control – cocci)) due to stable adhesion on large radii of curvature.

**Figure 8.**
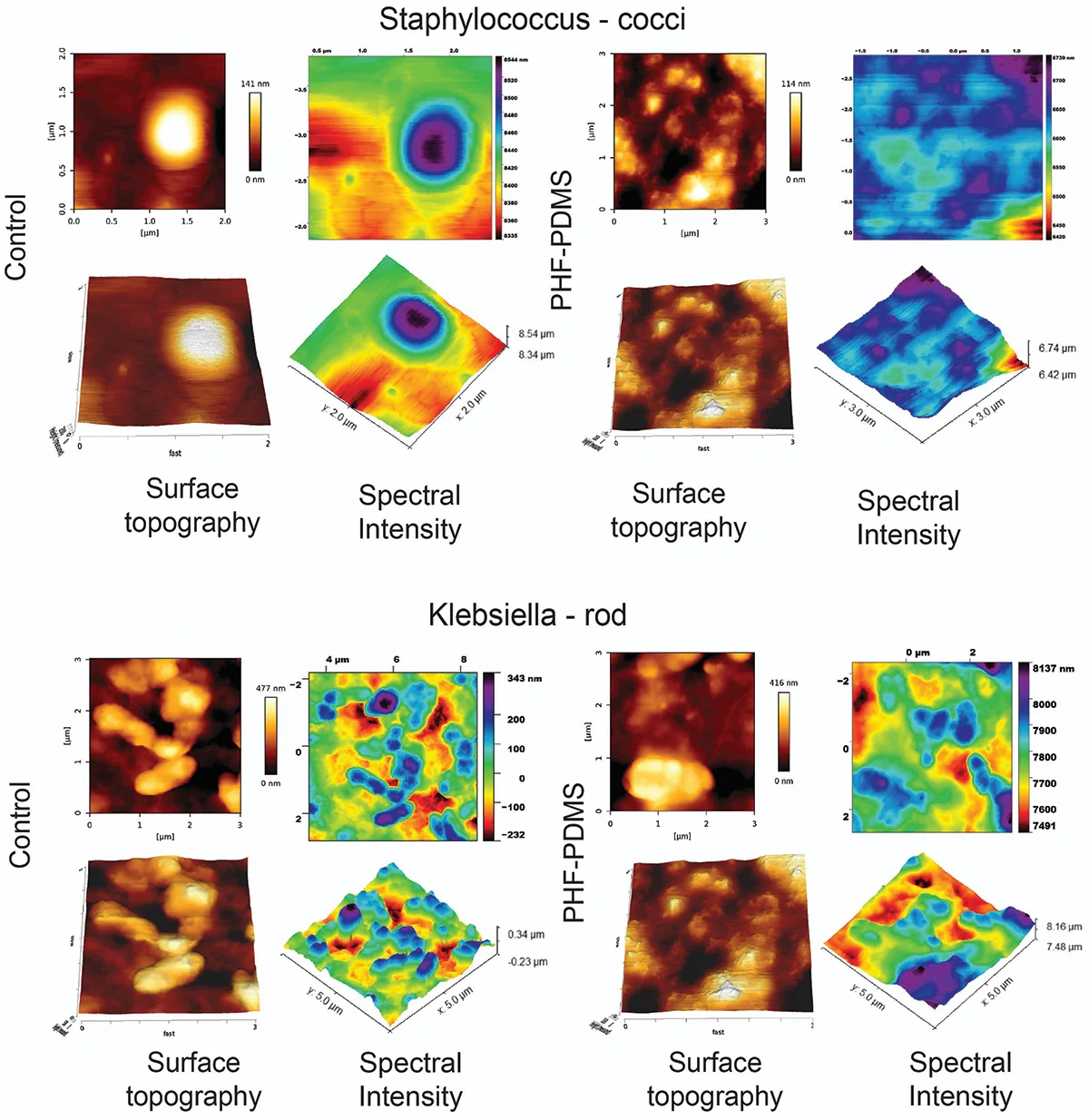
Topographical visualization of morphological modification of bacterial species grown on PHF-PDMS and pristine PDMS after overnight incubation.

In contrast, the PHF-PDMS surface of *Staphylococcus* cells exhibited heterogenous nanoscale morphology, marked by sharp asperities, and irregular domains with pronounced height variations. These profound height variations together with increased surface roughness is known to reduce surface activation energy making it an unfavourable for bacterial attachment [4]. Despite the increase in roughness, the bacterial adhesion behaviour seems to differ based on the roughness at microscale or nanoscale. Nanoscale roughness typically reduces bacterial adhesion by decreasing effective contact area and increasing localized mechanical stress, whereas microscale roughness may sometimes promote adhesion by providing sheltering sites for bacterial cells. This observation is consistent with other similar report [61,63].

Upon analysing the bacterial interaction with substrate, cocci exhibited smooth rounded edges on control, retaining their native morphology. This can be due to mechanically homogeneous nature of control PDMS surface at the nanoscale, which lacks stress-creating asperities and therefore allows uniform cell–surface contact without significant morphological deformation. Surface mechanics of treated surface revealed sharp topographical gradients dominated by blue-green domains (Figure 6) and localized asperities with a pronounced height variation of 250–350 nm. As a result, cocci grown on treated substrate showed stressed morphologies, including slight elongation and compression, indicating mechanical deformation induced by the underlying nanostructured surface roughness. This can be explained by the mechanically constrained nanoscale features of PHF-PDMS surface having high local curvature and enhanced asperity edges. A reduction in radii curvature corresponds to sharper asperities causing mechanical stress and non-uniform contact forces on bacterial cell membrane. Such localized stress results in altered morphology, including elongation or compression of cocci cells. Similar morphology-induced stress responses due to nanoscale asperities have been reported in an earlier study [67].

These mechanical barriers also reduce effective contact area between bacteria and the substrate causing reduced adhesion as confirmed by fluorescence staining. In the case of cocci structure, the morphological distortions exhibited were evident on observing the bacterial membrane showing crumbled cocci appearance. The reduced adhesion observed is likely due to reduced contact area dominated by peaks mostly [68].

Pronounced morphological alterations in the bacterial membrane and intracellular architecture were predominantly observed in the rod-shaped *Klebsiella* sp. Although both bacterial strains were exposed to similar substrate conditions, their surface interactions differed significantly due to their differences in cellular geometry. On the control substrate, rod-shaped cells retained their native elongated morphology, exhibiting smooth contours and a uniform height distribution along the longitudinal axis. This correlates with surface statistics of the control surface, demonstrating comparatively lower arithmetical mean height (Sa) and root mean square roughness (Sq) values, indicating reduced nanoscale irregularities.

Subsequently, such topographical characteristics on control favour extended line-contact adhesion between the bacterial cell wall and substrate, enabling uniform stress distribution across the cell membrane. Therefore, minimal surface distortion and preserved integrity of cell envelope were observed [62]. In converse, the PHF-PDMS exhibited positive S_sk_ values, suggesting the predominance of sharp surface peaks, while higher S_ku_ values (>3) indicated a leptokurtic height distribution characterized by extreme protrusions. These nanoscale roughness features promote localized point-contact adhesion rather than continuous line-contact interaction. As a result, mechanical stress becomes concentrated at discrete contact sites, generating significant membrane deformation. As a correspondent, rod bacilli on the PHF-PDMS surface displayed pronounced morphological disruption, including irregular terminal curvatures, fragmented elongation, membrane blebbing, and partial cellular collapse.

The constrained spreading observed may be attributed to mechanical stress imposed by high-curvature asperities and abrupt roughness transitions. This localized stress amplification can exceed the elastic tolerance of bacterial envelope, leading to membrane destabilization and compromised intracellular organization. These findings align with established mechano-bactericidal principles, wherein nanoscale topographical features induce mechanical rupture or severe deformation of bacterial membranes. The correlation between elevated roughness parameters (Sa, Sq), altered height distribution metrics (S_sk_, S_ku_), and observed cellular distortion strongly supports a topography-driven antibacterial mechanism [8,69].

The observed differences in morphological changes between cocci and rods, indicate that cellular geometry along with substrate roughness and skewness, significantly influences biomaterial-surface interactions. In the case of cocci, the contact points with substrate are relatively low and follow isotropic attachment. In contrast, rod structure exhibits anisotropic attachment, where contact occurs in longitudinal axis. This results in reduced focal points on curvature; the geometric difference between rod-shaped bacteria makes it more delicate to nanoscale asperities and curvature variations compared to cocci-shaped bacteria. Even on control topography of *Klebsiella*, on lower scan regions, rods exhibited interrupted or imperfect morphology, as it was dominated by sharp asperities. These findings strongly correlate with the other recent findings on rod bacteria and microcolony behaviour with the surface interaction [70,71]. While analysing PHF influence on bacteria, both cocci and rod exhibited membrane damage differing from control. In detail, for rod bacteria, analysis revealed smooth outer for control; whereas, PHF‑affected rod showed membrane damage and cellular leakage. Similarly, for cocci bacteria, PHF induced membrane crowding, leading to distorted outer membrane with dysmorphic morphology with marked inner blebbing (Figure 8). The fluorescence imaging results obtained from laser scanning microscopy, where cocci exhibited relatively higher surface coverage compared to rods on both control and PHF-PDMS substrates, further confirming the role of cellular geometry in bacterial adhesion patterns. Thereby increased fluorescence peaks observed for cocci on the PHF–PDMS substrate, but not for rods, may be attributed to increased asperity interactions causing mechanical intrusion into rod-bacteria, resulting in irreversible morphological changes that delay early proliferation and biofilm progression [72].

## 4. Conclusion

In conclusion, incorporation of PHF into PDMS significantly increased roughness of the substrate and thereby reducing bacterial adhesion. Modified surface effectively targets contact area by influencing surface mechanics and kinetics, leading to membrane damage and bactericidal activity. Fluorescent staining analysis revealed a marked reduction in bacterial attachment on PHF-PDMS compared to pristine PDMS for both *Klebsiella* and *Staphylococcus*. The PHF-PDMS exhibited markedly lower fluorescence intensity, fragmented adhesion patterns, and reduced spatial distribution, indicating impaired bacterial colonization. These results were in agreement with AFM-derived surface, wherein altered roughness, local curvature, and reduced contact areas disrupts bacteria–substrate interactions. Notably, a cellular geometry-dependent adhesion was evident from topography imaging, with rod-shaped *Klebsiella* exhibiting stronger adhesion inhibition than cocci-shaped *Staphylococcus* on PHF-PDMS. Behaviour is due to the sensitivity of elongated bacterial cells to nanoscale curvature and discontinuities, which limit the formation of stable, continuous contact interfaces. Collectively, the integration of fluorescence and surface mechanics findings establishes PHF-PDMS as an effective anti-adhesive surface, capable of restricting bacterial colonization at the initial attachment stage and thereby suppressing subsequent biofilm development on polymeric interfaces.
